# Addition of Inflammatory Biomarkers Did Not Improve Diabetes Prediction in the Community: The Framingham Heart Study

**DOI:** 10.1161/JAHA.112.000869

**Published:** 2012-08-24

**Authors:** Dhayana Dallmeier, Martin G. Larson, Na Wang, João D. Fontes, Emelia J. Benjamin, Caroline S. Fox

**Affiliations:** National Heart, Lung, and Blood Institute's Framingham Heart Study, Framingham, MA (D.D., M.G.L., J.D.F., E.J.B., C.S.F.); Division of General Internal Medicine, Boston University School of Medicine, Boston, MA (D.D.); Department of Cardiology, Boston University School of Medicine, Boston, MA (J.D.F., E.J.B.); Department of Biostatistics, Boston University School of Public Health, Boston, MA (M.G.L.); Department of Epidemiology, Boston University School of Public Health, Boston, MA (E.J.B.); Data Coordinating Center, Boston University School of Public Health, Boston, MA (N.W.); Department of Mathematics and Statistics, Boston University, Boston, MA (M.G.L.); Department of Endocrinology, Diabetes, and Metabolism, Brigham and Women's Hospital and Harvard Medical School, Boston, MA (C.S.F.)

**Keywords:** biomarkers, C-reactive protein, diabetes, inflammation, prediction

## Abstract

**Background:**

Prior studies have reported conflicting findings with regard to the association of biomarkers in the prediction of incident type 2 diabetes. We evaluated 12 biomarkers as possible diabetes predictors in the Framingham Heart Study.

**Methods and Results:**

Biomarkers representing inflammation (C-reactive protein, interleukin-6, monocyte chemoattractant protein-1, tumor necrosis factor receptor 2, osteoprotegerin, and fibrinogen), endothelial dysfunction (intercellular adhesion molecule-1), vascular damage (CD40-ligand, P-selectin, and lipoprotein-associated phospholipase A2 mass and activity), and oxidative stress (urinary isoprostanes) were measured in participants without diabetes attending the Offspring seventh (n=2499) or multiethnic Omni second (n=189) examination (1998–2001). Biomarkers were log_e_ transformed and standardized. Multivariable logistic regression tested each biomarker in association with incident diabetes at a follow-up examination (the Offspring eighth and Omni third examination; mean 6.6 years later), with adjustment for age, sex, cohort, body mass index, fasting glucose, systolic blood pressure, high-density lipoprotein cholesterol, triglycerides, and smoking. *C* statistics were evaluated with and without inflammatory markers. In 2638 participants (56% women, mean age 59 years), 162 (6.1%) developed type 2 diabetes. All biomarkers, excluding osteoprotegerin, were associated with the outcome with adjustment for age, sex, and cohort; however, none remained significant after multivariable adjustment (all *P*>0.05). The *c* statistic from the model including only clinical covariates (0.89) did not statistically significantly improve after addition of biomarkers (all *P*>0.10).

**Conclusions:**

Biomarkers representing different inflammatory pathways are associated with incident diabetes but do not remain statistically significant after adjustment for established clinical covariates. Inflammatory biomarkers might not be an effective resource to predict type 2 diabetes in community-based samples. **(*J Am Heart Assoc*. 2012;1:e000737 doi: 10.1161/JAHA.112.000869.)**

## Introduction

According to the Centers for Disease Control and Prevention, 25.6 million people were affected by diabetes in the United States in 2010, representing a 1.9-million–person increase over the previous year.^2^ The risk for death among people with diabetes is about twice that of people of similar age without diabetes, and the estimated costs for diabetes in the United States in 2007 were $174 billion, with $116 and $58 billion as direct and indirect medical costs, respectively.^3^

The importance of identifying individuals at high risk for the development of incident diabetes is supported by the results of the Diabetes Prevention Program, which showed that type 2 diabetes can be either prevented or delayed through lifestyle modifications and medications.^4–5^ Several different types of risk scores that incorporate clinical and nutritional variables have been developed around the world in an attempt to identify individuals at high risk for new-onset type 2 diabetes.^6–8^

The idea of a possible inflammatory etiology of diabetes originated >100 years ago, when high doses of sodium salicylate were effective in diminishing glycosuria among patients with the milder form of diabetes, most likely type 2 diabetes.^9^ This led to great interest in the concept that biomarkers of inflammation could improve the ability to identify individuals at risk for development of diabetes. However, previous studies evaluating the role of inflammatory biomarkers as possible predictors for the onset of type 2 diabetes in addition to the established clinical covariates reported conflicting findings.^10–20^ In addition, the majority of the studies demonstrating a valuable role for inflammatory biomarker(s) as predictors for the onset of type 2 diabetes were based on case–control studies, which not only lack the ability to establish temporality but also do not allow for a population-based assessment of risk-prediction metrics with and without the novel biomarker of interest. Thus, the purpose of the present study was to examine in a prospective cohort study, the Framingham Heart Study, whether inflammatory biomarkers improved the prediction of incident type 2 diabetes after adjustment for clinical covariates. We chose 12 biomarkers representing multiple inflammatory pathways, including inflammation (C-reactive protein [CRP], interleukin-6, monocyte chemoattractant protein-1, tumor necrosis factor receptor 2, osteoprotegerin, and fibrinogen), endothelial dysfunction (intercellular adhesion molecule), vascular damage (CD40-ligand, P-selectin, and lipoprotein-associated phospholipase A2), and oxidative stress (urinary isoprostanes).

## Methods

The present study was conducted in the Framingham Heart Study, a community-based, observational epidemiological cohort. The design and selection criteria of the Framingham Offspring study have been described.^21^ The Omni cohort, started in 1994, consisted of 506 men and women of African American, Hispanic, Asian, Indian, Pacific Islander, and Native American origins, who were residents of Framingham, MA, and surrounding towns at the time of enrollment. The baseline assessment for the present study involved Offspring participants at the seventh examination cycle (n=3539) and Omni participants at the second examination cycle (n=405). Participants were assessed for new-onset diabetes at the eighth follow-up examination for the Offspring and the third follow-up examination for the Omni participants. Participant exclusion criteria were as follows: prevalent type 2 diabetes at baseline (Offspring, n=366; Omni, n=53); off-site baseline examination (Offspring, n=205; Omni, n=2); missing baseline plasma glucose level (Offspring, n=3; Omni, n=69); incomplete information at baseline about baseline type 2 diabetes status, biomarkers, and clinical covariates (Offspring, n=17; Omni, n=2); failure to attend the subsequent examination (Offspring, n=416; Omni, n=78); and missing glucose data (Offspring, n=19; Omni, n=2) or incomplete diabetes status (Offspring, n=64; Omni, n=10) at the subsequent examination. Thus, a total of 2638 participants were included in the study (Offspring, n=2449; Omni, n=189). The study protocol was reviewed and approved by the Institutional Review Board of Boston University Medical Center. All participants gave written informed consent.

### Measurement of Inflammatory Biomarkers at Baseline Exam

Fasting samples were obtained at the seventh examination cycle for the Offspring participants and at the second examination cycle for the Omni participants, who had rested for 5 to 10 minutes in a supine position. Samples were stored and frozen at −80°C until testing. Serum CRP was measured with a high-sensitivity assay (Dade Behring BN100 nephelometer, Deerfield, IL). Commercially available ELISA methods (R&D Systems, Minneapolis, MN) were used to measure the serum concentrations of interleukin-6, intercellular adhesion molecule-1, and monocyte chemoattractant protein-1 and the plasma concentrations of CD40 ligand, osteoprotegerin, P-selectin, and tumor necrosis factor receptor 2. Plasma fibrinogen was measured by the Clauss method. Lipoprotein-associated phospholipase A2 mass and activity were measured by DiaDexus, Inc, San Francisco, CA. Urinary isoprostanes, 8-Epi-PGF_2α_, were indexed to urinary creatinine (ACE Competitive EIA – Cayman Chemical). All intra-assay coefficients of variation were ≤9.1%.

### Incident Type 2 Diabetes

New cases of type 2 diabetes were ascertained at the follow-up examination corresponding to examination cycle 8 for the Offspring and examination cycle 3 for the Omni (mean time of follow-up after baseline, 6.6 years; minimum 3.7, maximum 8.7 years). Type 2 diabetes was defined by a fasting glucose level ≥126 mg/dL or the use of insulin or oral hypoglycemic medications at the time of follow-up.

### Covariate Assessment

The covariates were defined at baseline examination through assessment of questionnaires, physicals, and laboratory tests. Current smoking status was classified by self-report of cigarette smoking during the year before examination. Blood pressure was measured twice at rest in a seated position by a physician using a mercury column sphygmomanometer, and measurements were averaged. Hypertension was defined as systolic blood pressure ≥140 mm Hg, diastolic blood pressure ≥90 mm Hg, or the use of antihypertensive medications. Body mass index was defined as the individual's body weight divided by height squared. Lipid profiles, plasma glucose, and insulin levels were measured from morning fasting blood samples with standardized assays. Impaired fasting glucose was defined as a fasting glucose level >100 mg/dL but <126 mg/dL. Aspirin use was defined as ≥3 doses per week.

### Statistical Analyses

Discrete variables are presented as counts and percentages, and continuous variables as means and standard deviations. The inflammatory markers' concentrations had skewed distributions and were natural log transformed. For comparable interpretations, the log biomarker concentrations were standardized (mean 0 and standard deviation 1). We estimated Pearson correlations among inflammatory biomarkers as well as between inflammatory biomarkers and clinical covariates.

We performed multivariable logistic regression with incident type 2 diabetes as the outcome. We evaluated the association of each individual inflammatory biomarker with the outcome, adjusting first for age, sex, and cohort (model 1). Then we added the following clinical covariates to model 1: body mass index, fasting glucose, systolic blood pressure, high-density lipoprotein cholesterol, triglycerides, and smoking (model 2). We estimated and compared the area under the curve between the baseline model including only clinical covariates and model 2 for each inflammatory biomarker. Differences in the *c* statistics were tested by the DeLong nonparametric method. The odds ratio is interpreted as the associated odds of developing diabetes per 1 standard deviation of the log biomarker level.

### Secondary Analyses

A test for effect modification by sex was performed for the inflammatory biomarkers with covariates from model 2. The available literature evaluating the role of CRP as a predictor for type 2 diabetes examined this association by comparing the upper quartiles with the lowest one.^10–20^ We evaluated the association between CRP and incident type 2 diabetes by performing a nonparametric analysis with splines, followed by an analysis of the association between sex-specific quartiles of CRP and the new onset of type 2 diabetes, with the use of model 1 and model 2.

For all analyses, we used SAS version 8.1 (SAS Institute, Cary, NC). For effect modification analyses, we established a significance level of *P*<0.01; for all other analyses we considered *P*<0.05 to be statistically significant.

## Results

Table 1 shows the participants' clinical characteristics at baseline, as well as by presence or absence of diabetes at the follow-up examination. There were 2638 participants (mean age 59±9 years, 56% women); most were white (93%). At baseline 890 (34%) individuals had impaired fasting glucose. At least 40% of the participants were receiving hypertension medications, lipid treatments, or aspirin at the time of follow-up. New-onset type 2 diabetes was detected in 162 participants at the follow-up examination (6.1% incidence). Among the new cases of type 2 diabetes, 121 were noted to have impaired fasting glucose at baseline. Those who developed diabetes in follow-up were more likely to be men and more likely to have been obese and to have had higher mean age and triglyceride levels, lower mean high-density lipoprotein concentrations, and higher prevalence of impaired fasting glucose, hypertension, or dyslipidemia at baseline. With regard to the inflammatory biomarkers, participants with incident diabetes had higher baseline levels of CRP, fibrinogen, interleukin-6, urinary isoprostanes, lipoprotein-associated phospholipase A2 mass, monocyte chemoattractant protein-1, P-selectin, and tumor necrosis factor receptor (Table 2). The reasons for exclusion and the clinical characteristics of those who did and did not attend the follow-up examination are presented in Tables 3 and 4**.** Participants not returning for follow-up were more likely to be ethnic or racial minorities, to have older mean ages, to have cardiovascular disease, and to have died in the interim.

**Table 1.** Participants' Characteristics at Baseline and According to Diabetes Status at Follow-Up

	Total (n=2638)	Diabetes (n=162)	No Diabetes (n=2476)
Age, y	59±9	62±9	59±9
Women, %	1479 (56)	69 (43)	1410 (57)
Race, n (%)			
White	2449 (93)	144 (89)	2305 (93)
African American	81 (3)	7 (4)	74 (3)
Hispanic	51 (2)	5 (3)	46 (2)
Asian	57 (2)	6 (4)	51 (2)
Omni, n (%)	189 (7)	18 (11)	171 (7)
Body mass index, kg/m^2^	27.7±5.1	31.5±5.6	27.5±4.9
Glucose, mg/dL	97±10	109±11	96±9
Triglycerides, mg/dL	129±80	180±136	125±73
High-density lipoprotein cholesterol, mg/dL	55±17	46±14	56±17
Systolic blood pressure, mm Hg	125±18	135±18	124±18
Impaired fasting glucose, n (%)	890 (34)	121 (75)	769 (31)
Hypertension, n (%)	1018 (39)	110 (68)	908 (37)
Hypertension treatment, n (%)	712 (27)	78 (48)	634 (26)
Lipid treatment, n (%)	444 (17)	48 (30)	396 (16)
Smoking, n (%)	306 (12)	18 (11)	288 (12)
Aspirin use, n (%)	725 (27)	62 (38)	663 (27)

Continuous variables are expressed as mean ± standard deviation.

Dichotomous variables are expressed as n (%).

**Table 2.** Biomarker Concentrations at Baseline According to Diabetes Status at Follow-Up

	Baseline (n=2638)	
	Diabetes (n=162)	No Diabetes (n=2476)
CRP, mg/L	3.51 (1.49, 8.15)	1.84 (0.90, 4.39)
CD40 ligand, ng/mL	1.21 (0.55, 4.04)	1.26 (0.54, 4.03)
Fibrinogen, mg/dL	382 (338, 445)	366 (324, 413)
Intercellular adhesion molecule-1, ng/mL	258 (223, 292)	236 (206, 273)
Interleukin-6, pg/mL	3.52 (2.27, 5.08)	2.38 (1.65, 3.74)
Urine isoprostanes, ng/mmol	147 (104, 204)	126 (87, 186)
LpaPA2 mass, nmol/mL per min	324 (268, 412)	302 (245, 371)
LpaPA2 activity, ng/mL	277 (216, 357)	282 (226, 352)
Monocyte chemoattractant protein-1, pg/mL	146 (122, 171)	138 (116, 163)
P-selectin, pg/mL	5.21 (4.50, 6.29)	5.15 (4.25, 6.13)
Osteoprotegerin, pmol/L	37 (30, 48)	35 (28, 44)
Tumor necrosis factor receptor 2, pg/mL	2100 (1804, 2570)	1888 (1586, 2256)

Values are given as median (lower quartile, upper quartile). CRP indicates C-reactive protein; LpaPA2, lipoprotein-associated phospholipase A2.

**Table 3.** Reasons for Participant Exclusion

	Baseline		Follow-Up	
Examination	Offspring 7	Omni 2	Offspring 8	Omni 3
Prevalent type 2 diabetes at baseline	366	53	…	…
Offsite baseline examination	205	2	…	…
Missing baseline plasma glucose level	3	69	…	…
Missing baseline type 2 diabetes status, biomarkers, and clinical covariates	17	2	…	…
Failure to attend subsequent examination	…	…	416	78
Missing glucose data on follow-up	…	…	19	2
Incomplete diabetes status on follow-up	…	…	64	10
Totals	591	126	499	90

**Table 4.** Participant Clinical Characteristics of Those Meeting Baseline Examination* Criteria by Status of Follow-Up Examination Attendance

	Attended Follow-Up Examination†		
Characteristics	Yes	No, Alive	No, Deceased
Baseline examination*, n	2733	291	203
Omni cohort, %	7	24	4
Female, %	56	58	39
Age, y	59.4	59.8	69.1
Prevalent cardiovascular disease, %	9	12	26
Glucose, mg/dL	96.6	96.9	99.5

* Offspring examination 7 or Omni examination 2.

† Offspring examination 8 or Omni examination 3.

### Relation of Individual Biomarkers With Incident Type 2 Diabetes

**Figure 1.**
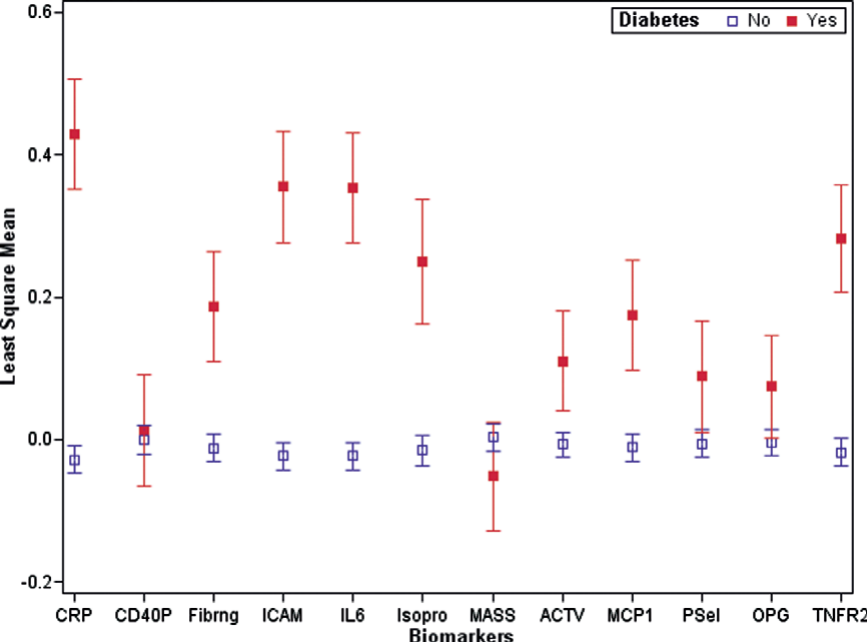
Least-squares adjusted means and standard errors for the different inflammatory biomarkers after adjustment for age, sex, and cohort. CRP indicates C-reactive protein; CD40L, CD40 ligand; ICAM, intercellular adhesion molecule 1; IL-6, interleukin-6; Isopro, urinary isoprostanes; MASS and ACTV, lipoprotein-associated phospholipase A2 mass and activity, respectively; MCP-1, monocyte chemoattractant protein-1; OPG, osteoprotegerin; Psel, P-selectin; and TNFR2, tumor necrosis factor receptor 2.

**Figure 2.**
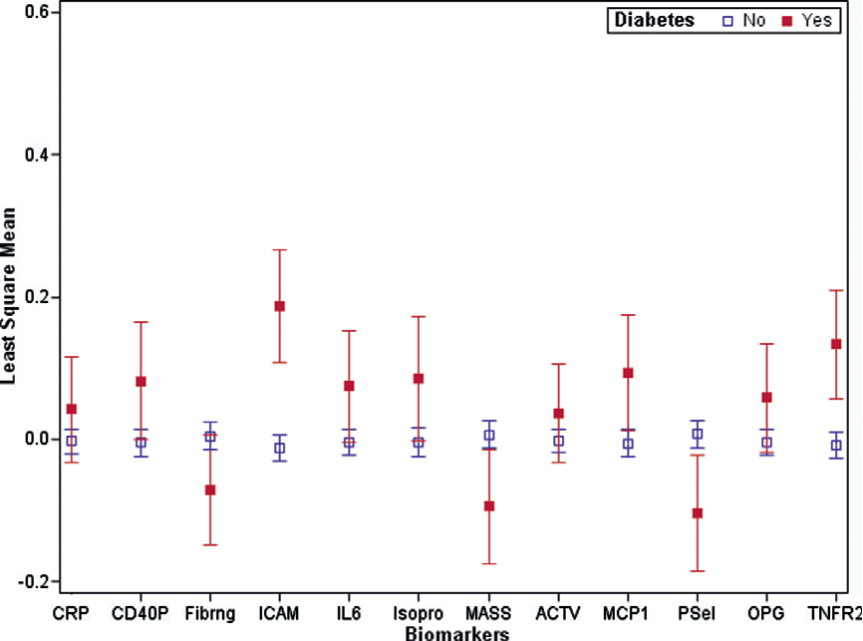
Least-squares adjusted means and standard errors for the different inflammatory biomarkers after adjustment for age, sex, cohort, body mass index, fasting glucose, systolic blood pressure, high-density lipoprotein cholesterol, triglycerides, and smoking. CRP indicates C-reactive protein; CD40L, CD40 ligand; ICAM, intercellular adhesion molecule 1; IL-6, interleukin-6; Isopro, urinary isoprostanes; MASS and ACTV, lipoprotein-associated phospholipase A2 mass and activity, respectively; MCP-1, monocyte chemoattractant protein-1; OPG, osteoprotegerin; Psel, P-selectin; and TNFR2, tumor necrosis factor receptor 2.

Adjusting for age, sex, and cohort, we found an association between the following 7 biomarkers and type 2 diabetes (all *P*<0.02): CRP, fibrinogen, intercellular adhesion molecule-1, interleukin-6, urinary isoprostanes, monocyte-chemoattractant protein-1, and tumor necrosis receptor factor 2 (Table 5 and Figure 1). The maximal statistically significant improvement of the area under the curve was observed upon addition of CRP (from 0.63 to 0.69, *P* value 0.002). However, once we additionally accounted for the clinical variables, all associations were attenuated (all *P*>0.07, Table 5 and Figure 2). The multivariable model containing clinical covariates showed an area under the curve equal to 0.89. We were not able to detect any improvement of the *c* statistic after addition of any of the inflammatory biomarkers (from 0.886 to 0.892, all *P* values >0.10 when compared to the clinical prediction model).

**Table 5.** Odds Ratios for Type 2 Diabetes Related to Biomarkers and *C* Statistics for the Corresponding Models

	Model 1: Age, Sex, Cohort				Model 2: Multivariable*			
Biomarkers†	OR (95% CI)	*P*	*C* Statistic	*P*‡	OR (95% CI)	*P*	*C* Statistic	*P*‡
Model without biomarkers			0.634				0.890	
CRP	1.64 (1.39–1.94)	<0.0001	0.692	0.002	1.17 (0.94–1.46)	0.16	0.891	0.34
CD40	1.02 (0.87–1.19)	0.85	0.633	0.43	1.06 (0.88–1.28)	0.51	0.891	0.29
Fibrinogen	1.24 (1.05–1.47)	0.011	0.648	0.20	0.91 (0.75–1.11)	0.35	0.890	0.68
ICAM	1.45 (1.24–1.70)	<0.0001	0.676	0.002	1.18 (0.99–1.42)	0.07	0.891	0.13
IL-6	1.42 (1.23–1.65)	<0.0001	0.677	0.003	1.07 (0.87–1.31)	0.55	0.890	0.38
Urine isoprostanes	1.33 (1.10–1.60)	0.003	0.641	0.044	1.16 (0.92–1.44)	0.21	0.886	0.22
LpaPA2 mass	0.95 (0.81–1.12)	0.55	0.633	0.79	0.92 (0.76–1.12)	0.41	0.890	0.73
LpaPA2 activity	1.17 (0.97–1.41)	0.10	0.638	0.51	1.14 (0.92–1.42)	0.24	0.892	0.14
MCP-1	1.23 (1.04–1.47)	0.018	0.644	0.35	1.17 (0.96–1.43)	0.13	0.891	0.11
P-selectin	1.11 (0.94–1.31)	0.23	0.637	0.43	0.89 (0.74–1.07)	0.21	0.891	0.71
Osteoprotegerin	1.09 (0.92–1.31)	0.32	0.637	0.30	0.98 (0.80–1.20)	0.86	0.890	0.71
TNFR2	1.36 (1.16–1.60)	0.0001	0.661	0.022	1.07 (0.88–1.30)	0.49	0.889	0.44

* Multivariable model: adjusted for age, sex, cohort, body mass index, fasting glucose, systolic blood pressure, high-density lipoprotein cholesterol, triglycerides, and smoking. Odds ratio (95% confidence interval) expressed as per standard deviation.

† CRP indicates C-reactive protein; CD40L, CD40 ligand; ICAM, intercellular adhesion molecule 1; IL-6, interleukin-6; LpaPA2, lipoprotein-associated phospholipase A2; MCP-1, monocyte chemoattractant protein-1; OPG, osteoprotegerin; and TNFR2, tumor necrosis factor receptor-2.

‡ *P* value for *c* statistic between model with biomarker and without biomarker.

### Secondary Analyses

There were no interactions with sex (all *P*>0.02). The additional analysis with sex-specific quartiles for CRP showed increased odds of developing diabetes among those in the third and fourth quartiles compared to the first quartile after adjustment for age, sex, and cohort. These associations were attenuated after addition of the clinical variables to model 1 (Table 6).

**Table 6.** Odds Ratios for Sex-Specific Quartiles for CRP in Relation to New-Onset of Type 2 Diabetes

	Model 1 (Adjusted for Age, Sex, and Cohort)		Model 2 (Multivariable Model*)	
	OR (95% CI)	*P*	OR (95% CI)	*P*
Second quartile vs lowest quartile	1.04 (0.57–1.88)	0.90	0.68 (0.35–1.30)	0.24
Third quartile vs lowest quartile	2.12 (1.25–3.59)	0.0050	1.04 (0.57–1.88)	0.91
Fourth quartile vs lowest quartile	3.31 (2.01–5.47)	<0.0001	1.21 (0.66–2.22)	0.54

* Multivariable model: adjusted for age, sex, cohort, body mass index, fasting glucose, systolic blood pressure, high-density lipoprotein cholesterol, triglycerides, and smoking.

We performed a post hoc power calculation. At a significance level of 0.05, we had 80% power to detect an association of type 2 diabetes with a biomarker, provided the true odds ratio was 1.30 to 1.38 per 1 standard deviation of the log biomarker level.

## Discussion

In our community-based sample, we observed an association between new-onset type 2 diabetes and biomarkers associated with inflammation (CRP, fibrinogen, interleukin-6, monocyte-chemoattractant protein-1, and tumor necrosis receptor factor 2), endothelial dysfunction (intercellular adhesion molecule), and oxidative stress (urinary isoprostanes) after adjustment for age, sex, and cohort. However, once we accounted for easily obtainable clinical predictors of type 2 diabetes, these biomarkers were no longer independent predictors of type 2 diabetes.

The experimental evidence suggesting a key role for inflammation in the pathogenesis of diabetes is compelling. Inflammation has been associated with insulin resistance through the activation of receptors and transcription factors that lead to β-cell dysfunction and apoptosis and impaired insulin signaling.^22^ The reduced sensitivity to insulin is believed to protect the organism in the initial stage of an inflammatory process, resulting in a better availability of substrate for the immune system. However, the risk of new onset of type 2 diabetes is increased by longstanding insulin resistance.^23^ In particular, obesity is considered one of the most important risk factors for the development of insulin resistance and type 2 diabetes. Macrophage cells recruited in the fat tissue release large amounts of proinflammatory cytokines, which result in an inhibitory effect on the insulin signaling.^24^

A sizable number of available studies have demonstrated an association between CRP, fibrinogen, interleukin-6, tumor necrosis factor-α, tumor necrosis factor-α receptor 2, and interleukin-18 and the new onset of diabetes.^10–20^ However, less has been reported about their ability to improve the discriminatory power of models containing well-established diabetes risk factors. Traditional statistical methods used for the assessment of etiological associations might not be adequate to determine the capacity of the marker of interest for classifying or predicting risk for the individual.^25^ In this setting, Salomaa et al^17^ identified in the FINRISK97 Cohort the biomarkers apolipoprotein B100, CRP, interleukin-1 receptor antagonist, and ferritin as the strongest predictors of incident diabetes. However, none of these biomarkers was able to improve the *c* index or the integrated discrimination improvement in the validation sample (Health 2000 cohort).

The Framingham type 2 diabetes clinical prediction model developed in 2007 showed that easily obtainable clinical characteristics could adequately predict type 2 diabetes, with a *c* statistic of 0.89. Indeed, the primary risk factors for developing type 2 diabetes were fasting glucose levels, the presence of obesity (body mass index ≥30 kg/m^2^), high-density lipoprotein cholesterol levels, evidence for a positive parental history, triglyceride levels ≥150 mg/dL, and elevated blood pressure after adjustment for age and sex.^8^ Evaluating each of the inflammatory biomarkers in a model containing only well-established clinical covariates, our results failed to show an association between any of the biomarkers and the new onset of type 2 diabetes but also did not provide an improvement in the area under the curve of the baseline model based on the clinical covariates. Given the lack of association and the lack of a substantial change in the *c* statistic, we do not have any strong indication that the selected biomarkers could improve the ability to separate individuals who will go on to develop diabetes from those who will not.^26^ Therefore, the calculation of the net reclassification improvement and the integrated discrimination improvement were not warranted in this study.

### Strengths and Limitations

The strengths of the present study include a community-based sample, routine ascertainment of the outcome of interest and potential confounders, and the availability of a robust set of inflammatory markers, which were measured serially with precise techniques to quantify their concentrations. Some limitations warrant mention. Our sample represents mostly non-Hispanic white individuals, and therefore the generalizability of our findings to other ethnic or racial minority groups is uncertain. Similarly, we did not have sufficient numbers of any one ethnic or racial group to perform separate analyses. The results of our analysis were based on single-occasion biomarker measurements, so day-to-day variability of the biomarker itself could not be assessed. New cases of type 2 diabetes were identified through a single measurement of fasting glucose levels and not through an oral glucose tolerance test as recommended by the World Health Organization.^27^ Whereas a challenge test could be important pathophysiologically, oral glucose tolerance tests are not used in everyday clinical practice and are unlikely to be a major source of bias in our work. Our analysis does not include parental history of diabetes, which could contribute to a higher *c* statistic on the baseline model. However, following the obtained results, one would not expect an improvement of the area under the curve of such a baseline model after addition of each individual biomarker. In addition, we acknowledge some selection and survival biases in our longitudinal assessment. We had modest power to detect a small effect of the association of interest. Whereas the modest power might have affected our ability to detect small effects, it is unlikely that such effects would lead to substantial improvement in discrimination.

Systemic inflammation is associated with incident type 2 diabetes. We acknowledge that it is possible that inflammation contributes to the development of increasing blood pressure and fasting glucose, which could be in the causal pathway. However, none of the 12 inflammatory biomarkers remained associated with incident type 2 diabetes after standard clinical diabetes risk factors were accounted for, and the addition of the inflammatory biomarkers did not provide a significant improvement in the area under the curve. Our results reinforce the messages that easily obtainable clinical predictors are sufficient to identify individuals at risk for the development of diabetes and that inflammatory biomarker assessments are unlikely to be resource effective in further risk-stratification of individuals at risk of new-onset diabetes.
